# Effects of Virtual Reality on Motor Function and Balance in Incomplete Spinal Cord Injury: A Systematic Review and Meta-Analysis of Controlled Trials

**DOI:** 10.3390/brainsci15101071

**Published:** 2025-09-30

**Authors:** Yamil Liscano, Florencio Arias Coronel, Darly Martínez

**Affiliations:** Grupo de Investigación en Salud Integral (GISI), Departamento Facultad de Salud, Universidad Santiago de Cali, Cali 760035, Colombia; florencio.arias00@usc.edu.co (F.A.C.); darly.martinez00@usc.edu.co (D.M.)

**Keywords:** virtual reality, augmented reality, spinal cord injury, motor function, balance, neurorehabilitation, systematic review, meta-analysis

## Abstract

**Background/Objectives**: Incomplete spinal cord injury (iSCI) represents a significant challenge in neurorehabilitation, with conventional limitations including recovery plateaus and declining patient motivation. Virtual reality (VR) and augmented reality (AR) have emerged as promising technologies to supplement traditional therapy through gamification and multisensory feedback. This systematic review and meta-analysis evaluates the effectiveness of VR and AR interventions for improving balance and locomotor function in patients with incomplete spinal cord injury. **Methods**: A systematic review was conducted following PRISMA guidelines, with searches in PubMed, Scopus, Web of Science, Science Direct, and Google Scholar. Randomized controlled trials and high-quality controlled studies evaluating VR/AR interventions in patients with iSCI (American Spinal Injury Association Impairment Scale [AIS] classifications B, C, or D) for a minimum of 3 weeks were included. A random-effects meta-analysis (Standardized Mean Difference, SMD; 95% Confidence Interval, CI) was conducted for the balance outcome. **Results**: Eight studies were included (*n* = 142 participants). The meta-analysis for balance (k = 5 studies) revealed a statistically significant improvement with a large effect size (SMD = 1.21, 95% CI: 0.04–2.38, *p* = 0.046). For locomotor function, a quantitative meta-analysis was not feasible due to a limited number of methodologically homogeneous studies; a qualitative synthesis of this evidence remained inconclusive. Substantial heterogeneity was observed in the balance analysis (I^2^ = 81.5%). No serious adverse events related to VR/AR interventions were reported. **Conclusions**: VR/AR interventions show potential as an effective adjunctive therapy for improving balance in patients with iSCI, though the benefit should be interpreted with caution due to considerable variability between studies. The current evidence for locomotor function improvements is insufficient to draw conclusions, highlighting a critical need for more focused research. Substantial heterogeneity indicates that effectiveness may vary according to specific intervention characteristics, populations, and methodologies. Larger multicenter studies with standardized protocols are required to establish evidence-based clinical guidelines.

## 1. Introduction

Spinal cord injury (SCI) represents a major global health challenge, with annual incidence rates reaching up to 80 cases per million people worldwide. These injuries, stemming from both traumatic events and non-traumatic pathologies, inflict devastating and permanent damage to the central nervous system, leading to severe impairments in motor, sensory, and autonomic function [1,2]. For individuals with incomplete spinal cord injury (iSCI), the preservation of some neural pathways offers significant potential for functional recovery. However, this patient population still faces a complex array of chronic obstacles, including impaired mobility, persistent pain, muscle spasticity, and considerable psychosocial distress that can impede their rehabilitation progress and overall quality of life (QoL) [3,4].

The cornerstone of care for iSCI is conventional neurorehabilitation, which focuses on structured, repetitive exercises to restore strength, coordination, and functional independence. While this approach is fundamental, its effectiveness can be constrained by several factors. Progress often slows or reaches a plateau after an initial period of recovery [4], and the highly repetitive, often clinical nature of the exercises can lead to a decline in patient motivation and engagement over the extended course of therapy [5,6]. This lack of sustained, active participation is a critical barrier to maximizing long-term functional outcomes [7,8].

In response to these limitations, immersive technologies such as virtual reality (VR) and augmented reality (AR) have emerged as innovative tools to supplement traditional rehabilitation. VR technology immerses a user in a completely simulated, computer-generated environment, while AR overlays digital information onto the user’s view of the real world [9]. By leveraging gamification and providing interactive, goal-directed tasks, these technologies transform therapeutic exercise into an engaging and motivating experience. They allow for the safe practice of complex activities, like navigating obstacles or maintaining balance in challenging scenarios, that would be difficult or unsafe to replicate in a standard clinical setting [10,11].

The therapeutic efficacy of VR and AR is primarily attributed to their ability to drive neuroplasticity—the brain’s innate capacity to reorganize itself in response to experience. These technologies facilitate motor relearning by providing high-repetition, task-specific practice coupled with immediate, multi-sensory feedback, which are key principles for inducing positive neural changes [12]. The adaptability of these systems allows clinicians to precisely tailor the intensity and complexity of the virtual tasks to each patient’s individual needs and progress, ensuring an optimal level of challenge to foster continuous improvement [13,14,15].

Despite the growing body of research and promising preliminary findings, the clinical translation of VR and AR is hindered by considerable heterogeneity across studies. Research in this field employs a wide variety of technologies, intervention protocols, session durations, and outcome measures, making it difficult to establish clear, evidence-based conclusions about overall effectiveness [16,17]. A rigorous synthesis of the available evidence is therefore essential to guide clinical practice. This systematic review and meta-analysis aims to consolidate and evaluate the evidence from controlled trials to determine the effectiveness of VR and AR interventions for improving balance and locomotor function in patients with incomplete SCI.

## 2. Materials and Methods

This systematic review was conducted in accordance with the Preferred Reporting Items for Systematic Reviews and Meta-Analyses (PRISMA) guidelines [18]. The methodology was designed to ensure a comprehensive and unbiased selection of relevant studies evaluating the effectiveness of VR and augmented reality in managing balance and locomotor function in patients with incomplete SCI.

Research Question (PICO): In patients with incomplete SCI (P), how does VR or augmented reality supplementation, or the supplementation of both (I), compared to conventional therapy or standard treatments (C), affect balance OR locomotor function, thereby influencing functional mobility and QoL (O)?

This study’s Prospero registration number is CRD420251102611.

### 2.1. Eligibility Criteria

The eligibility criteria for this review are detailed in Table 1 below.

### 2.2. Information Sources and Search Strategy

A comprehensive search of electronic databases was conducted to identify relevant studies. The databases searched included the following:

PubMed; Science Direct; Scopus; Web of Science; Google Scholar.

The data were organized using Zotero version 7.0 (accessed on 10 July 2025).

### 2.3. Search Algorithm

The search strategy combined Medical Subject Headings (MeSH) and free-text terms related to SCI, VR, augmented reality, motor function, and balance. Boolean operators (AND, OR) were used to refine the search. An example of the search strategy used in PubMed is as follows:

(“spinal cord injury” OR “spinal cord lesion” OR “incomplete spinal cord injury” OR “paraplegia” OR “tetraplegia” OR “SCI”) AND (“virtual reality” OR “augmented reality” OR “mixed reality” OR “VR” OR “AR” OR “immersive technology” OR “extended reality” OR “XR”) AND (“motor function” OR “motor recovery” OR “balance” OR “postural control” OR “functional mobility” OR “rehabilitation” OR “neurorehabilitation” OR “locomotor training” OR “Berg Balance Scale” OR “ASIA Motor Score”)

This search algorithm was adapted for each database according to its specific search functionalities. Additional searches were conducted by reviewing the reference lists of relevant articles to identify any studies that might have been missed.

### 2.4. Study Selection

Two reviewers (D.M. and F.A.) independently screened the titles and abstracts of all identified articles for eligibility. Full-text articles were retrieved for studies that appeared to meet the inclusion criteria or if eligibility was unclear from the abstract. Discrepancies between the reviewers were resolved through discussion, and if necessary, a third reviewer (Y.L.) was consulted to reach a consensus.

### 2.5. Data Extraction

Two reviewers (D.M. and F.A.) independently extracted information from the primary studies using a standardized data extraction form. The extracted data included details of the clinical trial:

- Study characteristics: first author, publication year, country, and study design.
- Participant characteristics: number of participants, age, BMI, sex, and diagnostic criteria for SCI.
- Intervention details: type of VR/augmented reality used, technical characteristics, dosage, form of administration, and duration of intervention.
- Comparator: details of the control group (placebo or standard care).
- Outcomes measured: primary outcomes (e.g., motor function markers, balance parameters), secondary outcomes (e.g., QoL, inflammatory markers), and methods of measurement.
- Results: main findings, statistical significance, and conclusions drawn by the authors.
- Limitations: as reported by the authors.

Subsequently, a third reviewer (Y.L.) verified the integrity and accuracy of the recorded information.

The flow diagram was created with the online R package PRISMA2020 [18] (https://estech.shinyapps.io/prisma_flowdiagram/, accessed on 10 July 2025), while the data visualizations were produced using the R software package ggplot2, version 4.4.1 (https://cran.r-project.org/bin/windows/base/old/4.4.1/, accessed on 10 July 2025).

### 2.6. Risk-of-Bias Assessment

The risk-of-bias assessment for the included studies was conducted independently by two reviewers (Y.L. and F.A.) using the Cochrane Risk of Bias Tool for Randomized Trials [19], with data entered into Review Manager version 5.4^®^ (RevMan; The Cochrane Collaboration, London, UK). Available online: https://training.cochrane.org/online-learning/core-software-cochrane-reviews/revman (accessed on 10 July 2025). The following domains were evaluated:

- Random sequence generation (selection bias)
- Allocation concealment (selection bias)
- Blinding of participants and personnel (performance bias)
- Blinding of outcome assessment (detection bias)
- Incomplete outcome data (attrition bias)
- Selective reporting (reporting bias).

In addition to the risk-of-bias analysis, the methodological quality of the RCTs was assessed using the Jadad scale [20,21]. This 5-point scale evaluates the quality of trials based on their reporting of randomization, blinding, and the management of withdrawals and dropouts.

For non-randomized controlled studies, the following domains were evaluated using the Risk Of Bias In Non-randomized Studies—of Interventions (ROBINS-I) tool [22]: Bias due to confounding; Bias in selection of participants; Bias in classification of interventions; Bias due to deviations from intended interventions; Bias due to missing data; Bias in measurement of outcomes; Bias in selection of the reported result.

For each domain, studies were assessed as having a low, high, or unclear risk of bias based on predetermined guidelines. Discrepancies in the risk-of-bias assessment were resolved through discussion between the reviewers, and if necessary, a third reviewer (D.M.) was consulted.

To confirm the consistency of the evaluation process, a subset of the studies was re-assessed, and Cohen’s kappa coefficient was computed using IBM SPSS Statistics version 29.0 (IBM Corp., Armonk, NY, USA) to quantify the agreement between reviewers.

### 2.7. Data Synthesis

Due to the heterogeneity among the included studies in terms of interventions, technologies used, dosages, and outcomes measured, both qualitative and quantitative syntheses were conducted when appropriate. The results are presented in narrative form, accompanied by tables summarizing the key characteristics and findings of the studies.

Statistical Analysis: Meta-analysis was performed using R software version 4.4.1 (R Foundation for Statistical Computing, Vienna, Austria) with the following packages: ‘meta’ (version 7.0-0) for conducting meta-analyses, ‘metafor’ (version 4.6-0) for advanced meta-analytic procedures, and ‘ggplot2’ (version 3.5.1) for creating publication-quality visualizations. All analyses were conducted on 10 July 2025. A quantitative meta-analysis was performed for balance outcomes. For locomotor function outcomes, a qualitative synthesis was conducted due to significant clinical heterogeneity between studies.

Two primary outcomes were analyzed:

- Balance outcomes (5 studies, *n* = 125 participants): A meta-analysis was conducted on studies reporting outcomes such as the modified Functional Reach Test (mFRT), Limits of Stability (LOS), and postural sway measurements.
- Locomotor function outcomes: A qualitative synthesis was performed on studies evaluating lower extremity motor recovery (e.g., Lower Extremity Motor Score) and gait. A meta-analysis was not conducted due to the limited number of homogeneous studies (k = 2), which was deemed insufficient for a robust quantitative analysis.

For the balance meta-analysis, standardized mean differences (SMD) with 95% confidence intervals (CIs) were calculated for continuous outcomes using Hedges’ g to correct for small sample bias. The inverse variance method was employed for pooling effect sizes. Both common effect and random effects models were fitted, with the restricted maximum-likelihood (REML) estimator used for calculating between-study variance (τ^2^). The Hartung–Knapp adjustment was applied to obtain more conservative CIs in the random effects model.

Heterogeneity was assessed using the Q statistic and quantified with I^2^ statistics, where values of 25%, 50%, and 75% were considered as low, moderate, and substantial heterogeneity, respectively.

Forest plots were generated to visualize individual study effects and the pooled estimate for the balance outcome. A funnel plot was created to assess publication bias for this outcome; however, due to the limited number of studies (k = 5 for balance), formal testing for publication bias using Egger’s test was not conducted, as it requires a minimum of 10 studies for reliable results. A funnel plot was not generated for locomotor function, as no meta-analysis was performed.

Sensitivity analyses were conducted using a leave-one-out approach to evaluate the robustness of the pooled estimates for the balance outcome.

Subgroup analysis based on the level of VR immersion was planned. However, due to the limited number of studies within each potential subgroup for the balance outcome, a formal analysis of subgroup differences was not conducted to avoid over-interpreting the data. All statistical tests were two-tailed with a significance level set at *p* < 0.05.

Given the heterogeneity in measurement instruments within each outcome category (e.g., different balance scales and units), standardized mean differences were essential to allow for meaningful comparison across studies.

### 2.8. Ethical Considerations

As this study is a systematic review of the published literature, it did not involve direct interaction with human subjects or animals and thus did not require ethical approval.

## 3. Results

### 3.1. Studies Identified for the Review

The systematic database search initially identified 1239 records. The distribution across databases was as follows: PubMed (*n* = 735), Scopus (*n* = 207), Web of Science (*n* = 147), Science Direct (*n* = 100), and Google Scholar (*n* = 50). After the removal of 201 duplicates, 1038 articles remained for the initial screening phase.

The screening process demonstrated high inter-rater reliability, with a Cohen’s kappa coefficient of 0.91 for the title and abstract review. Based on the eligibility criteria applied during this stage, 1023 articles were excluded. Subsequently, the full texts of the remaining 15 articles were retrieved for detailed eligibility assessment. This second stage also showed substantial agreement between reviewers, with a Cohen’s kappa of 0.89.

The full-text review resulted in the exclusion of 8 additional studies. The reasons for exclusion were: three were review articles or meta-analyses, three did not meet the required methodological design (i.e., they were not randomized controlled trials or high-quality controlled studies), and two were excluded because their intervention duration was less than three weeks.

**Figure 1:**
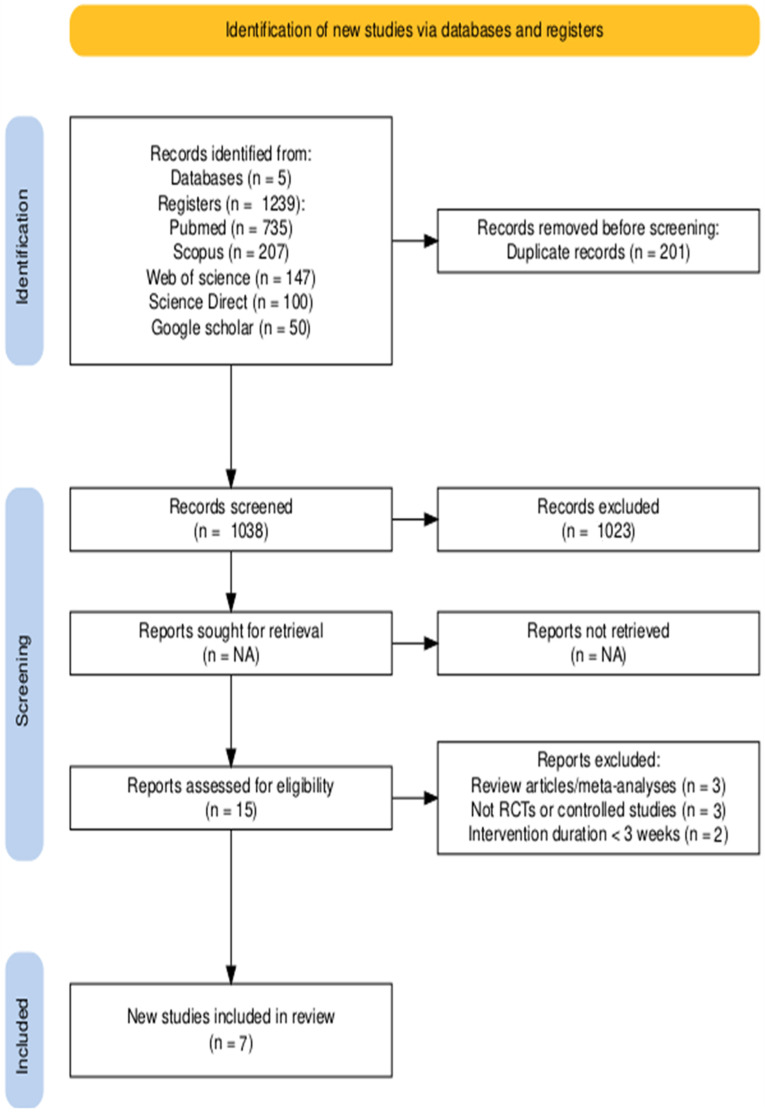
PRISMA flow diagram with the search and study selection strategy. The inter-rater agreement for study selection was substantial, with Cohen’s Kappa coefficients of 91% for title and abstract screening and 89% for full-text eligibility assessment, indicating high consistency between reviewers in the selection process. The abbreviation ‘NA’ (not applicable) indicates fields that were not relevant to our search process or represents a default output from the software used to generate the diagram.

Ultimately, 7 studies met all inclusion criteria and were included in the final systematic review and meta-analysis. These studies featured high-quality experimental designs, appropriate target populations (patients with incomplete SCI, AIS grades B, C, or D), relevant interventions using virtual or augmented reality, adequate duration (≥3 weeks), and assessment of the prespecified outcomes. The complete study selection process is illustrated in the PRISMA flow diagram (Figure 1).

### 3.2. Characteristics of Included Studies

The seven studies included in this review were published between 2015 and 2024 and represent research from diverse geographical regions. The distribution included three studies from South Korea and one each from The Netherlands, Switzerland, The United States, and India. A summary of the characteristics of each included study is provided in Table 2.

### 3.3. Characteristics of Participants and Interventions

#### 3.3.1. Demographic and Clinical Characteristics

The total study population comprised 142 participants with incomplete SCI. The mean age of the participants varied across studies, ranging from 39.1 to 67 years. A general predominance of male participants was observed, with male-to-female ratios ranging from 5:0 to 10:3. Detailed demographic and clinical data for the participants in each study are presented in Table 3.

#### 3.3.2. Characteristics of VR Interventions

The VR interventions across the included studies were considerably heterogeneous in terms of the technology used, level of immersion, and application protocols. The implemented technologies ranged from non-immersive systems, such as the Nintendo Wii, to immersive systems like the GRAIL platform or head-mounted displays such as Ocular grand VR spectacles. The specific characteristics of each VR intervention are detailed in Table 4.

### 3.4. Risk of Bias and Methodological Quality

#### 3.4.1. Risk of Bias in RCTs

**Figure 2:**
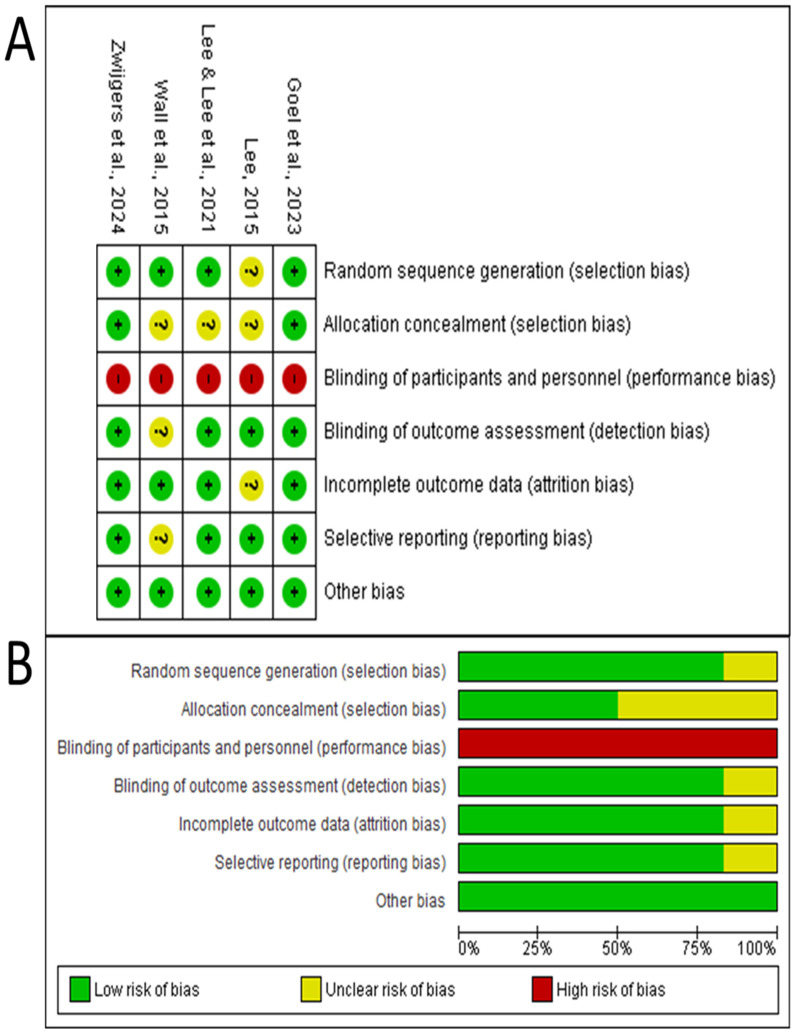
Risk of bias evaluation for the randomized controlled trials included in this review using the Cochrane Risk of Bias Tool (RoB 2). (**A**) Individual study assessments showing risk of bias across six domains for each RCT. The “+” symbol (green) denotes low risk of bias, “?” (yellow) indicates unclear risk, and “–” (red) represents high risk of bias [23,24,25,27,29]. (**B**) Summary of risk of bias assessment across all six RCTs, displaying the percentage distribution of low risk (green), unclear risk (yellow), and high risk (red) for each bias domain. Note that all studies showed high risk for blinding of participants and personnel due to the inherent nature of VR interventions, which represents an unavoidable limitation in this field of research.

The risk of bias analysis using the Cochrane RoB 2 tool revealed that all RCTs had a high risk of bias in the domain of participant and personnel blinding (Figure 2). This is an inherent and unavoidable limitation in this field of research due to the overt nature of VR interventions. In contrast, the domain of random sequence generation was predominantly rated at low risk (83.3% of studies). Allocation concealment showed greater variability, with 50% of studies rated as having an unclear risk of bias.

#### 3.4.2. Methodological Quality via Jadad Scale

The assessment using the Jadad scale yielded an average score of 3.2 out of 5 (range: 2–4), indicating a moderate overall methodological quality for the included RCTs (Table 5). Notably, two studies achieved a score of 4, signifying good quality, as they demonstrated adequate randomization procedures, appropriate descriptions of withdrawals, and proper blinding of outcome assessment.

#### 3.4.3. ROBINS-I Assessment for Non-Randomized Studies

The risk of bias assessment for the two non-randomized controlled studies, conducted using the ROBINS-I tool, revealed that both studies were rated as having a low overall risk of bias (Table 6).

### 3.5. Qualitative Synthesis of Results

#### 3.5.1. Balance Outcomes

All seven included studies evaluated the effects of VR interventions on balance function. The findings were consistently positive, showing improvements across various aspects of balance performance. For instance, Lee and Lee (2021) [23] found that their VR group demonstrated statistically significant improvements in both Force Sensitive Application (FSA) and Limit of Stability (LOS) measures compared to the control group (*p* < 0.05). Similarly, An and Park (2018) [28] reported that participants receiving semi-immersive VR therapy showed significant gains in overall LOS scores (from 32.00 to 46.40, *p* < 0.01) and Berg Balance Scale (BBS) scores (from 35.70 to 40.10, *p* < 0.01). Furthermore, Wall et al. (2015) [27] demonstrated that training with the Nintendo Wii Fit led to significant improvements in forward functional reach (*p* < 0.001) and lateral functional reach (*p* = 0.001), with effects maintained at a 4-week follow-up.

#### 3.5.2. Motor Function Outcomes

Two studies assessed changes in locomotor function parameters following VR supplementation: Villiger et al. (2017) [26] and Zwijgers et al. (2024) [24] targeted lower extremity and locomotor function. Villiger et al. (2017) [26] reported significant improvements in the Lower Extremity Motor Score (LEMS) following a home-based VR training program (*p* = 0.008). Due to the small number of studies assessing these specific outcomes, a quantitative synthesis was not feasible.

#### 3.5.3. Functional Mobility Outcomes

Four studies evaluated functional mobility using standardized measures such as the Timed Up and Go (TUG) test and the Walking Index for SCI-II (WISCI-II). The results showed mixed but generally positive trends. An and Park (2018) [28], for example, found significant improvements in TUG performance, with times decreasing from 19.35 to 17.14 s (*p* < 0.05). Additionally, WISCI-II scores in their study improved significantly from 16.30 to 17.90 (*p* < 0.05), suggesting a reduced reliance on assistive devices.

#### 3.5.4. QoL and Secondary Outcomes

The assessment of QoL as a specific outcome was notably absent in the studies included in this review. None of the seven trials that met the final inclusion criteria utilized standardized instruments to measure QoL. This highlights a significant gap in the current literature regarding the impact of VR interventions on this crucial patient-reported outcome.

#### 3.5.5. Adverse Events and Safety

Six of the seven studies commented on the safety profile of VR supplementation. None of these trials reported any serious adverse events related to the VR interventions. Villiger et al. (2017) [26] noted that no participants experienced pain or spasticity during training sessions and reported high motivation scores. Similarly, An and Park (2018) [28] explicitly stated that no participants experienced cybersickness or other VR-related side effects during their semi-immersive training.

### 3.6. Quantitative Synthesis: Meta-Analysis Results

#### 3.6.1. Effects on Balance

**Figure 3:**
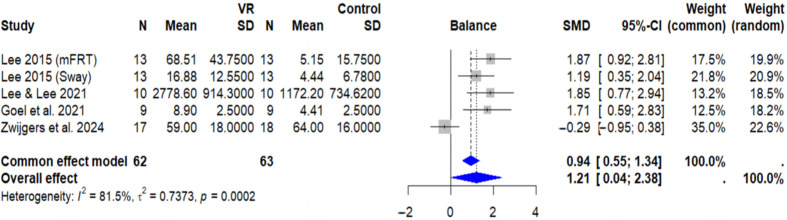
Forest plot showing the effects of VR on balance. The random-effects model (SMD = 1.21, 95% CI: 0.04–2.38, *p* = 0.046) demonstrates a statistically significant overall effect. However, substantial heterogeneity was detected (I^2^ = 81.5%, *p* < 0.001). The inverse variance method was used with a restricted maximum-likelihood estimator for τ^2^ and the Hartung–Knapp adjustment for the random-effects model. Abbreviations: SMD, standardized mean difference; CI, confidence interval [23,24,25,29].

The meta-analysis for balance outcomes included 5 studies with a total of 125 participants. The results demonstrated a large and statistically significant effect of VR interventions on balance. The random-effects model yielded a standardized mean difference (SMD) of 1.21 (95% CI: 0.04–2.38, *p* = 0.046), indicating a strong positive effect of VR on balance measures (Figure 3).

However, this result must be interpreted with caution. The CI is wide, and its lower bound is very close to the line of no effect (0.04), suggesting that while the finding is statistically significant, its robustness could be limited. Furthermore, substantial heterogeneity was observed among the studies (I^2^ = 81.5%, *p* < 0.001), indicating considerable variability in treatment effects across the different trials.

#### 3.6.2. Assessment of Publication Bias for Balance Outcomes

**Figure 4:**
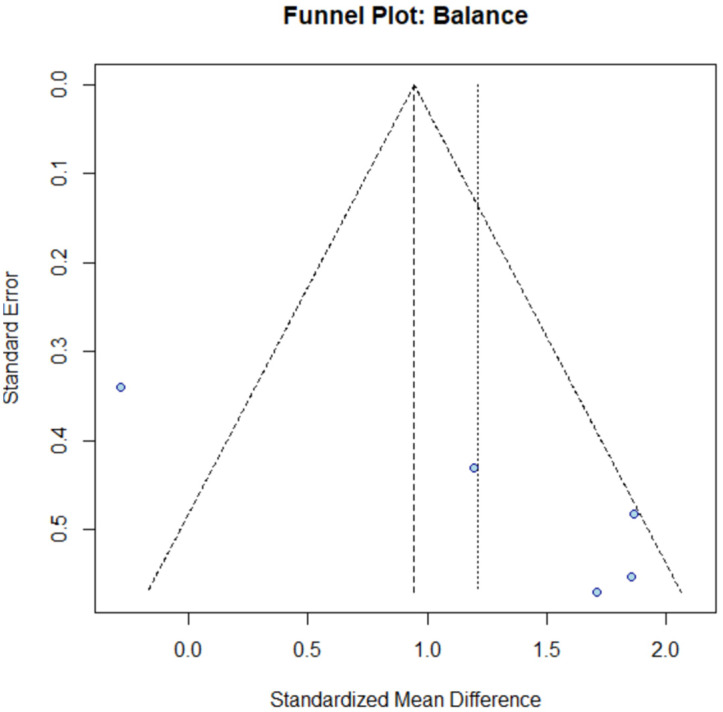
Funnel plot for the assessment of publication bias in balance studies. The asymmetrical distribution of studies is likely attributable to the substantial heterogeneity (I^2^ = 81.5%) rather than publication bias. The limited number of studies (k = 5) precludes a reliable formal assessment of publication bias [23,24,25,29].

To investigate the potential for publication bias, a funnel plot was generated (Figure 4). Visual inspection of the plot, which maps study precision (standard error) against effect size (SMD), reveals some asymmetry in the distribution of the five studies. Specifically, there is considerable scatter in the effect sizes. One study with low precision (high standard error) is located to the left of the pooled estimate, near the line of no effect, while several other low-precision studies cluster on the far right, indicating very large effects.

This asymmetrical pattern is highly consistent with the substantial statistical heterogeneity (I^2^ = 81.5%) already detected, as genuine variability in outcomes across studies can distort the plot’s symmetry. Furthermore, the plot suggests the presence of a “small-study effect,” where smaller, less precise studies tend to report larger effect sizes than larger, more precise studies. Given the limited number of studies (k = 5), formal statistical tests for funnel plot asymmetry are unreliable. Therefore, while asymmetry is noted, it should be interpreted with caution and is likely influenced more by the high heterogeneity than by publication bias.

## 4. Discussion

### 4.1. Main Findings

The primary objective of this systematic review and meta-analysis was to evaluate the efficacy of VR interventions as adjunctive therapy for rehabilitation in patients with incomplete SCI. Following the analysis of seven studies comprising 142 participants, the quantitative synthesis revealed statistically significant improvements in balance function, with a large effect size (SMD = 1.21, 95% CI: 0.04–2.38, *p* = 0.046). However, for locomotor function outcomes, a meta-analysis was not performed due to the limited number of studies assessing this outcome (k = 2), which was deemed insufficient for a robust quantitative analysis. The qualitative synthesis of the available evidence for locomotor function remains inconclusive.

It is crucial to emphasize the substantial heterogeneity observed for balance outcomes, with an I^2^ value of 81.5%. This high degree of variability indicates considerable differences among studies in factors such as technology implementation, immersion levels, intervention protocols, participant characteristics, and outcome measurement methods. The findings suggest that treatment effects may vary significantly depending on specific intervention characteristics, population features, or methodological approaches, thereby limiting the generalizability of the pooled estimates.

Despite the statistical significance observed for balance outcomes, the very wide CI, with a lower bound close to the line of no effect (0.04), and substantial between-study variability indicate that the magnitude of benefit may be highly variable across different clinical contexts. Individual studies demonstrated positive effects across various balance measures, including Force Sensitive Application, Limit of Stability, Berg Balance Scale, and functional reach tests. However, the diversity of assessment tools and interventions used across studies makes it challenging to determine which specific combinations of technology, protocols, and populations yield optimal outcomes.

Regarding safety profiles, six of the seven studies reported no serious adverse events related to interventions, suggesting that these technologies are generally well-tolerated in the incomplete SCI population. This finding supports the feasibility of implementing VR-based rehabilitation strategies, though longer-term safety data and larger sample sizes would strengthen this conclusion.

The limited number of studies available for the meta-analysis (five for balance) precluded a reliable assessment of publication bias, as formal statistical tests require a minimum of 10 studies. The observed asymmetry in the funnel plot for balance appears to be more attributable to the substantial heterogeneity than to selective publication of results. An analysis of publication bias for locomotor function was not applicable as no meta-analysis was conducted.

### 4.2. Comparison with Previous Literature

The findings of this meta-analysis demonstrate both convergence and divergence with previous research in rehabilitation for neurological populations. The significant effect on balance function (SMD = 1.21) aligns with a recent systematic review by Wang et al. (2024) [30] that evaluated 16 studies on VR in SCI, reporting significant improvements in gait capacity measured by the Walking Index for SCI (WISCI) with a mean difference of 1.29 points (95% CI: 0.07 to 2.51; *p* = 0.04) and significant increases in the limit of stability (LOS) with SMD = 1.75 (95% CI: 0.99 to 2.52; *p* < 0.01) [30]. This concordance strengthens the evidence that VR interventions can generate clinically relevant benefits in postural control.

The comprehensive systematic review by Scalise et al. (2024) [31], which analyzed 46 studies with 652 SCI patients, provides additional perspective on VR effectiveness. This study reported that no trial demonstrated inferior results for VR compared to traditional therapy, and in comparative studies, 71% of lower limb mobility, balance, and gait measures favored VR over traditional training. These findings support our observations of beneficial effects on balance, although the substantial heterogeneity (I^2^ = 81.5%) observed in our analysis suggests that the incomplete SCI population may present unique challenges in terms of intervention standardization and response variability.

Regarding motor function outcomes, our finding that the evidence is inconclusive due to a lack of sufficient homogeneous studies aligns with the results of Wang et al. (2024) [30] who also reported no significant improvements in lower limb motor function (Lower Extremity Motor Score; MD = 2.60; 95% CI: −1.58 to 6.79; *p* = 0.22) nor in activities of daily living evaluated with SCIM (*p* > 0.16 in all sub-scores). This concordance suggests that the motor benefits of VR in SCI may be more subtle or require more specific protocols than the balance benefits.

A recent exploratory study by Rosiak et al. (2022) [32] that validated commercial VR headsets as standalone posturographic devices provides relevant mechanistic insights. The study demonstrated that immersive VR significantly increased postural sway at the head level, with increases of 4.2 times in mild perturbation conditions and 8.8 times during storm simulations (*p* < 0.001). These findings suggest that immersive virtual environments generate sensory-vestibular conflicts that may be therapeutically useful for balance training, providing a mechanistic basis for the effects observed in our meta-analysis.

### 4.3. Proposed Mechanisms of Action in SCI Rehabilitation

The theoretical foundations for VR effectiveness in incomplete SCI rehabilitation are grounded in principles of neuroplasticity and motor learning theory. There are four primary neuroplastic mechanisms through which VR can benefit patients:

#### 4.3.1. Mirror Neuron System Activation and Virtual Embodiment

VR-based mirror therapy leverages the mirror neuron system by reflecting movements of an intact limb, thereby “tricking” the brain into activating motor pathways of the affected side. The visual reappearance of self-actions in the VR scene stimulates the activity of affected cortical areas and promotes their functional integration. VR-based motor imagery exercises may increase cortical mapping of areas corresponding to the muscle being trained and the excitability of the corticospinal tract, facilitating motor relearning [33,34,35].

#### 4.3.2. Cortical Reorganization Through Multisensory Stimulation

This principle is due to cross-modal plasticity via multisensory stimulation. By concurrently engaging visual, auditory, and proprioceptive systems, VR can create a rich sensory experience that fosters synaptic reorganization in specific locations that depend on the type of training and VR system utilized [36]. Wang et al. (2024) [30] reported that Villiger et al. [26] found that VR can improve functional outcomes in SCI patients by enhancing structural brain plasticity at cortical and brainstem levels following training.

#### 4.3.3. Error-Based Learning and Real-Time Adaptive Feedback

Advanced VR platforms can capture real-time kinematic data, allowing for immediate feedback and task adjustment. This closed-loop system can reflect principles of motor learning by reinforcing correct movements and discouraging maladaptive patterns [37]. Evidence suggests that such feedback can facilitate the strengthening of residual pathways and accelerate recovery. The findings of Rosiak et al. (2022) [32] support this mechanism by demonstrating that transient visual perturbations modulate electrocortical activity and enhance short-term postural learning.

#### 4.3.4. Reward Mechanisms and Cognitive Engagement

Gamification and immersive scenarios can stimulate dopaminergic pathways, especially those in the ventral striatum, crucial for motivation and learning. The interactive, goal-oriented nature of VR can increase patient adherence while improving cognitive functions such as attention, memory, and executive control [38,39].

#### 4.3.5. Sensory-Vestibular Integration and Postural Control

The sensory-vestibular conflicts generated by immersive VR environments represent a specific mechanism for balance improvement. Rosiak et al. (2022) [32] demonstrated that commercial VR headsets induce vestibule-ocular conflict and elicit measurable vestibule-spinal reflexes. The disparity between immersive visual stimuli and vestibular signals forces the nervous system to develop more robust postural control strategies, explaining the improvements observed in balance function in our meta-analysis.

#### 4.3.6. Brain-Gut Axis Modulation and Neuroimmunomodulatory Response

Although not directly studied in the context of VR rehabilitation, recent research has highlighted the importance of the brain-gut axis in neurological recovery. The stress-reduction and mood-enhancement effects of immersive virtual experiences may indirectly influence systemic inflammatory processes and recovery through neuroendocrine pathways [40,41,42]. The psychological outcomes review by Williamson et al. (2025) [43] in SCI reported that VR interventions significantly reduced depressive and anxiety scores, suggesting systemic effects that may contribute to functional recovery.

### 4.4. Limitations of the Included Studies

The findings of this systematic review must be interpreted within the context of several important methodological and practical limitations identified in the analyzed studies. First, the relatively small sample sizes across most trials, ranging from 5 to 41 participants with an average of 20.3 participants per study, significantly compromises the statistical power to detect clinically meaningful differences in outcomes such as functional mobility or QoL. This limitation is particularly problematic for studies like Wall et al. (2015) [27], which included only 5 participants, making it impossible to draw meaningful conclusions about intervention effectiveness.

The heterogeneity of interventions represents a major limitation that complicates interpretation of results. Studies employed technologies ranging from non-immersive gaming systems (Nintendo Wii) to fully immersive systems such as the GRAIL platform or head-mounted displays like Ocular grand VR spectacles, with vastly different levels of immersion, interaction capabilities, and sensory feedback. Session durations varied from 30 to 60 min, with frequencies ranging from 2 to 5 times per week, and total intervention periods spanning 4 to 8 weeks. This diversity in intervention parameters makes it challenging to determine optimal dosing protocols or to identify which specific characteristics contribute most to therapeutic benefits.

Methodological quality concerns further limit the reliability of findings. The risk of bias analysis revealed that all randomized controlled trials had high risk of bias in participant and personnel blinding, an unavoidable limitation given the nature of interventions. However, additional concerns included unclear allocation concealment in 50% of studies and inadequate description of randomization procedures in some trials. The average Jadad score of 3.2 out of 5 indicates only moderate methodological quality, with one study (Wall et al., 2015) [27] receiving a poor quality rating.

The diversity of outcome measures used across studies presents another significant limitation. Balance outcomes were assessed using various instruments including Force Sensitive Application, Limit of Stability, Berg Balance Scale, modified Functional Reach Test, and postural sway measures. The diversity of outcome measures for locomotor function (e.g., Lower Extremity Motor Score) and the small number of studies assessing it were key factors preventing a pooled analysis.

The limited follow-up periods in most studies (ranging from 4 to 8 weeks) preclude assessment of long-term effects or durability of interventions. Given that neuroplasticity and motor learning processes may require months to years to fully manifest, the short-term nature of these studies may underestimate the true potential of rehabilitation. Only one study (Wall et al., 2015) [27] included a follow-up assessment (4 weeks post-intervention), which is insufficient to determine lasting effects.

Population characteristics also present limitations for generalizability. The included studies showed considerable variability in participant characteristics, including age ranges (39.1 to 67 years), time since injury (ranging from days to years), lesion levels (cervical, thoracic, lumbar), and AIS grades (A, B, C, D). Most studies focused on chronic SCI patients, limiting applicability to acute or subacute populations. The predominance of male participants (observed male-to-female ratios ranging from 5:0 to 10:3) further limits generalizability to female patients.

Geographic and cultural limitations are evident, as a significant portion (three of seven studies) of the included studies were conducted in South Korea, with the remainder carried out in India, the Netherlands, Switzerland, and the United States. This concentration in a limited number of regions may restrict the generalizability of the findings to populations with different healthcare systems, cultural perspectives on technology, or genetic backgrounds.

### 4.5. Limitations of the Review

The conclusions of this review must be considered in light of several methodological limitations. First, the search strategy was restricted to studies published in English, which may have introduced a language bias and led to the omission of relevant research from non-Anglophone regions.

Second, substantial statistical heterogeneity was a major challenge throughout the analysis. For the balance outcomes, the high heterogeneity (I^2^ = 81.5%) indicates that the observed positive effect is not uniform across all studies. This variability complicates the formulation of specific clinical recommendations, as the magnitude of the benefit likely depends on factors not fully elucidated by our analysis. For locomotor function, a quantitative synthesis was not performed simply due to the scarcity of available evidence, with only two studies meeting the inclusion criteria that focused on this outcome.

Third, this review is limited by the outcomes reported in the primary studies. A comprehensive meta-analysis of key clinical outcomes, such as QoL, activities of daily living (ADL), and long-term functional status, was not possible. Notably, none of the seven included studies reported on QoL using a standardized instrument, highlighting a critical gap in the literature regarding patient-centered outcomes.

The review’s scope, defined by its inclusion criteria, may have excluded other valuable sources of information. The requirement for a minimum intervention duration of three weeks, while intended to capture lasting effects, might have omitted studies on intensive, short-term protocols. Additionally, the exclusive focus on controlled trials, while maximizing internal validity, prevents the analysis of observational data that could offer insights into the real-world implementation and effectiveness of VR technologies in diverse clinical settings.

### 4.6. Clinical Implications

The findings of this review have several important implications for clinical practice. The integration of VR technology represents a promising adjunctive approach in SCI rehabilitation, capable of enhancing conventional therapy programs, particularly for balance training.

For balance function, the large and statistically significant effect size (SMD = 1.21) suggests that VR interventions can provide meaningful clinical benefits. Clinicians can consider implementing VR as a tool to improve postural control, leveraging its potential to increase patient engagement and training intensity in a safe, controlled environment. However, this recommendation must be tempered by two key findings: the high heterogeneity and the wide CI of the effect (95% CI: 0.04–2.38). This indicates that while the average effect is positive, clinical outcomes are likely to be highly variable. Therefore, clinicians should not expect uniform success and must be prepared to personalize protocols and closely monitor individual patient responses, as the benefit could be marginal in some cases.

For locomotor function, the current evidence is insufficient to guide routine clinical implementation. The inability to conduct a meta-analysis, due to a scarcity of studies with homogeneous methodologies and conflicting results among them, means there is no clear evidence base to support the use of VR for improving lower extremity motor recovery. Clinicians should view the application of VR for this purpose as experimental and prioritize it only after further high-quality research emerges.

Ultimately, the successful implementation of VR in clinical practice requires a personalized approach. The variability in patient response underscores the need for individualized assessment and treatment planning. While the safety profile of VR appears favorable based on the included studies, practitioners should remain vigilant for potential side effects like cybersickness. Finally, the integration of these technologies demands careful consideration of resource allocation, including the cost of equipment and the necessity for specialized staff training to maximize therapeutic benefit [44,45].

### 4.7. Recommendations for Future Research

Future research in VR rehabilitation for SCI should prioritize large-scale, multi-center randomized controlled trials with adequate sample sizes to definitively establish intervention effectiveness and identify optimal protocols. Studies should include a minimum of 100 participants per group to achieve adequate statistical power for detecting clinically meaningful differences in primary outcomes.

The standardization of intervention protocols represents a critical research priority. Future studies should systematically investigate the effects of different parameters including immersion level, session duration, frequency, and total intervention duration. Comparative effectiveness research directly comparing different technologies (non-immersive vs. immersive systems) and intervention approaches would help to establish evidence-based guidelines for clinical implementation [46].

Scalise et al. (2024) [31] highlight the need to integrate artificial intelligence and machine learning algorithms into VR rehabilitation systems to provide personalized adaptive programs that adjust difficulty in real-time. This research direction, combined with the development of portable wearable systems with wireless sensors, could facilitate home-based rehabilitation and tele-rehabilitation models.

Long-term effectiveness research is essential to determine whether intervention benefits are sustained over time and whether repeated training cycles provide additional benefits. Research should also investigate the optimal timing of interventions within the continuum of SCI rehabilitation, from acute care through long-term community reintegration.

Mechanistic studies investigating the neurophysiological effects of VR interventions are needed to better understand how these technologies promote recovery in SCI. Neuroimaging studies using functional MRI, EEG, or other techniques could provide insights into the neural mechanisms underlying improvements and identify biomarkers predictive of treatment response [7,47,48].

Finally, implementation science research is needed to understand the barriers and facilitators to VR adoption in clinical practice. Studies should examine factors such as healthcare provider attitudes, training requirements, technological infrastructure needs, and patient acceptance to develop strategies for successful program implementation across diverse healthcare settings [44,45].

## 5. Conclusions

This systematic review and meta-analysis provides evidence supporting the use of virtual reality and augmented reality technologies as adjunctive therapies in the rehabilitation of patients with incomplete spinal cord injury, particularly for balance training. The results demonstrate that VR/AR interventions can generate clinically relevant improvements in balance function with a large effect size, suggesting potential advantages over conventional rehabilitation methods through enhanced motivation, increased training intensity, and the ability to provide consistent, repeatable challenges in controlled environments.

However, the substantial heterogeneity observed across studies emphasizes the need for personalization in clinical implementation and careful consideration of factors such as technology selection, intervention protocols, and individual patient characteristics. Current evidence for locomotor function improvements remains inconclusive, not due to a lack of effect, but due to a paucity of homogeneous studies suitable for meta-analysis, requiring further research to identify optimal protocols. The absence of reported serious adverse events supports the feasibility of implementing VR-based rehabilitation strategies in clinical practice, though longer-term safety data with larger samples are needed. Future research should prioritize large-scale, multicenter randomized controlled trials with adequate sample sizes, standardized intervention protocols, and long-term effectiveness evaluation to establish evidence-based clinical guidelines and optimize the integration of these promising technologies into standard neurorehabilitation care.

## Figures and Tables

**Table 1:** Eligibility criteria.

Criteria	Inclusion	Exclusion
Study Design	- Randomized controlled trials (RCTs) investigating supplementation with virtual reality (VR), augmented reality (AR), or combined technologies in patients with incomplete SCI—Controlled non-randomized studies (quasi-experimental designs) with appropriate control groups and high methodological quality.	- Observational studies, reviews, cohort studies, case series, or uncontrolled studies. - Studies without an appropriate control group.
Participants	- Patients diagnosed with incomplete SCI according to the American Spinal Injury Association Impairment Scale (AIS) classifications B, C, or D. - Studies with mixed populations (complete and incomplete SCI) that report separate outcome data for AIS B, C, or D participants. - Adults approximately 18–65 years of age.	- Patients with concomitant neurological conditions.
Interventions	- Supplementation with VR, AR, or combined technologies administered in any form (e.g., immersive systems, semi-immersive platforms, portable devices).	- Supplementation with technologies other than VR or AR (e.g., conventional therapy alone without VR/AR integration).
Duration	- Interventions with a minimum duration of 3 weeks to adequately assess changes in motor and balance parameters.	- Interventions with a duration of less than 3 weeks.
Outcomes	- Studies evaluating effects on motor function (e.g., ASIA Motor Score, modified Ashworth Scale), balance (e.g., Berg Balance Scale, postural stability index), or functional mobility, as well as changes in other relevant markers (e.g., quality of life, neuropathic pain).	- Studies that only evaluated clinical outcomes such as respiratory function without assessing motor function, balance, or functional mobility. - Studies that focused exclusively on upper extremity motor function without assessing balance or locomotor function.
Language	- Studies published in English.	- Studies published in languages other than English.

Abbreviations: AIS, American Spinal Injury Association Impairment Scale; AR, augmented reality; ASIA, American Spinal Injury Association; RCT, randomized controlled trial; SCI, spinal cord injury; VR, virtual reality.

**Table 2:** Characteristics of the studies included in the review.

Author, Year	Country	Study Design	Population	Number of Patients	Outcomes Evaluated	Duration (Weeks)
Lee and Lee, 2021 [23]	South Korea	RCT	AIS C, D	20	Balance (FSA, LOS)	8
Zwijgers et al., 2024 [24]	Netherlands	RCT	AIS C, D	41	Motor function, balance, functional mobility	6
Lee, 2015 [25]	South Korea	RCT	AIS A, B	26	Balance (postural sway, mFRT, T-shirt test)	6
Villiger et al., 2017 [26]	Switzerland	Controlled study	AIS C, D	12	Motor function, balance, functional mobility	4
Wall et al., 2015 [27]	United States	RCT	AIS D	5	Balance, gait, functional mobility	7
An and Park, 2018 [28]	South Korea	Controlled study	AIS C, D	10	Balance (LOS, BBS), functional mobility	6
Goel et al., 2023 [29]	India	RCT	Incomplete paraplegia	28	Balance (sitting balance control)	4

Abbreviations: AIS: American Spinal Injury Association Impairment Scale; RCT: randomized controlled trial; FSA: Force Sensitive Application; LOS: Limit of Stability; mFRT: modified Functional Reach Test; BBS: Berg Balance Scale.

**Table 3:** Characteristics of the study populations.

Study	Mean Age (Years)	Sex (M/F)	Lesion Level	AIS	Time Post-Injury
Lee 2015 [25]	VR: 49.5 ± 8.3, C: 43.1 ± 11.2	VR: 10/3, C: 10/3	VR: 4C/9T, C: 5C/8T	VR: 10A/3B, C: 10A/3B	VR: 21.7 ± 8.7 m, C: 22.4 ± 9.4 m
Zwijgers et al., 2024 [24]	VR: 62 (56–71), C: 67 (60–72)	VR: 10/7, C: 9/9	Mixed (Cervical, Thoracic, Lumbar)	C, D	VR: 47 (20–120) m, C: 66 (20–135) m
Wall et al., 2015 [27]	58.6 (range 50–64)	5/0	Mixed (C4, C5, C6, L1)	D	>1 year (chronic)
An and Park, 2018 [28]	44.2 ± 8.7	6/4	Mixed (8 Cervical/2 Thoracic)	C, D	19.2 ± 3.9 months
Goel et al., 2023 [29]	VR: 41.9 ± 5.8, C: 39.1 ± 9.1	VR: 7/2, C: 8/1	Not specified (Paraplegia)	B, C, D	VR: 7.6 ± 1.2 m, C: 6.9 ± 1.1 m
Lee and Lee, 2021 [23]	VR: 55.1 ± 10.4, C: 53.7 ± 6.6	VR: 9/1, C: 4/6	Thoracic/Lumbar	C, D	VR: 16.5 ± 4.7 m, C: 17.4 ± 5.1 m
Villiger et al., 2017 [26]	60 ± 10.2	10/2	Mixed (C4-L3)	C, D	>1 year (chronic)

Abbreviations: AIS, American Spinal Injury Association Impairment Scale; C, control group; C, cervical; F, female; L, lumbar; M, male; m, months; T, thoracic; VR, virtual reality group.

**Table 4:** VR intervention characteristics.

Study	VR Technology	Immersion Type	Duration/ Session	Frequency	Total Sessions	Activities/Games
Lee 2015 [25]	Nintendo Wii	Non-immersive	30 min	3×/week	18	Tennis, table tennis, boxing, golf, bowling, frisbee, canoe, swordplay
Zwijgers et al., 2024 [24]	GRAIL (Motek Medical B.V.)	Immersive (VR environment)	60 min (20 min active)	~2×/week	11	Gait adaptability tasks: precision stepping, obstacle avoidance, reacting to perturbations
Wall et al., 2015 [27]	Nintendo Wii Fit	Non-immersive	60 min	2×/week	14	Games to promote weight shifting and balance (Penguin plunge, Segway, Island bike, etc.)
An and Park, 2018 [28]	IREX (GestureTek)	Semi-immersive	30 min	3×/week	18	Soccer, conveyor, volleyball, formula racer, airborne, snowboard
Goel et al., 2023 [29]	Ocular grand VR spectacles	Immersive	45 min	5×/week	20	Roller Coaster VR, In Mind VR, VR Tunnel Race
Lee and Lee, 2021 [23]	Bio Rescue (RM Ingenierie)	Semi-immersive	30 min	3×/week (implied)	24 (implied)	Rally driving, air balloon, downhill ski
Villiger et al., 2017 [26]	YouKicker (YouRehab AG)	Immersive (1st person view)	30–45 min	4–5×/week	16–20	Hamster Splash, Footbag, Get to the Game, Star Kick, Planet Drive

Abbreviations: VR, virtual reality. GRAIL (Motek Medical B.V., Amsterdam, The Netherlands); IREX (GestureTek, Ottawa, ON, Canada); Bio Rescue (RM Ingenierie, Rodez, France); YouKicker (YouRehab AG, Schlieren, Switzerland).

**Table 5:** Methodological quality assessment using the Jadad scale for RCTs.

Study	Randomized (0–1)	Double-Blind (0–1)	Withdrawals Described (0–1)	Randomization Adequate (0–1)	Blinding Adequate (0–1)	Total Score (0–5)	Quality Level
Lee and Lee, 2021 [23]	1	0	1	1	0	3	Moderate
Zwijgers et al., 2024 [24]	1	0	1	1	1	4	Good
Lee, 2015 [25]	1	0	1	0	1	3	Moderate
Goel et al., 2023 [29]	1	0	1	1	1	4	Good
Wall et al., 2015 [27]	1	0	0	1	0	2	Poor

Average Jadad Score: 3.3/5; Quality levels: Poor (≤2), Moderate (3), Good (4–5).

**Table 6:** Risk of bias assessment using ROBINS-I for non-randomized controlled studies.

Study	Bias Due to Confounding	Bias in Selection of Participants	Bias in Classification of Interventions	Bias Due to Deviations	Bias Due to Missing Data	Bias in Measurement of Outcomes	Bias in Selection of Reported Results	Overall Risk
Villiger et al., 2017 [26]	Low	Low	Low	Low	Low	Low	Low	Low
An and Park, 2018 [28]	Low	Low	Low	Low	Low	Low	Low	Low

## Data Availability

The data are contained within the article.

## Abbreviations

The following abbreviations are used in this manuscript:

SCI	Spinal cord injury
iSCI	Incomplete spinal cord injury
VR	Virtual reality
AR	Augmented reality
AIS	American Spinal Injury Association Impairment Scale
ASIA	American Spinal Injury Association
RCT	Randomized controlled trial
PRISMA	Preferred Reporting Items for Systematic Reviews and Meta-Analyses
PICO	Patient/Population, Intervention, Comparison, Outcome
MeSH	Medical Subject Headings
SMD	Standardized mean difference
CI	Confidence interval
REML	Restricted maximum-likelihood
FSA	Force Sensitive Application
LOS	Limit of Stability
mFRT	Modified Functional Reach Test
ASIA-UEMS	American Spinal Injury Association Upper Extremity Motor Score
WHOQOL-BREF	World Health Organization Quality of Life-Brief
BBS	Berg Balance Scale
TUG	Timed Up and Go
WISCI-II	Walking Index for Spinal Cord Injury-II
QoL	Quality of life
LEMS	Lower Extremity Motor Score
ABC	Activity-specific Balance Confidence
SCIM	Spinal Cord Independence Measure
XR	Extended reality
ROBINS-I	Risk Of Bias In Non-randomized Studies—of Interventions
RevMan	Review Manager
